# Impact of BTV-3 Circulation in Belgium in 2024 and Current Knowledge Gaps Hindering an Evidence-Based Control Program

**DOI:** 10.3390/v17040521

**Published:** 2025-04-03

**Authors:** Virginie Van Leeuw, Ilse De Leeuw, Nicolas Degives, Pieter Depoorter, Jeroen Dewulf, Jean-Baptiste Hanon, Jozef Hooyberghs, Annick Linden, Laura Praet, Marc Raemaekers, Claude Saegerman, Xavier Simons, Charlotte Sohier, Norbert Steurbaut, Amandine Sury, Etienne Thiry, Stephan Zientara, Axel Mauroy, Nick De Regge

**Affiliations:** 1 Federal Agency for the Safety of the Food Chain, 1000 Brussels, Belgium; pieter.depoorter@favv-afsca.be (P.D.); jozef.hooyberghs@favv-afsca.be (J.H.); laura.praet@favv-afsca.be (L.P.); marc.raemaekers@favv-afsca.be (M.R.); amandine.sury@favv-afsca.be (A.S.); axel.mauroy@favv-afsca.be (A.M.); 2 National Reference Laboratory for Bluetongue, Service of Exotic and Vector-Borne Diseases, Sciensano, 1080 Brussels, Belgium; charlotte.sohier@sciensano.be (C.S.); nick.deregge@sciensano.be (N.D.R.); 3 Coordination of Veterinary Activities and Veterinary Epidemiology, Sciensano, 1080 Brussels, Belgium; nicolas.degives@sciensano.be (N.D.); jean-baptiste.hanon@sciensano.be (J.-B.H.); xavier.simons@sciensano.be (X.S.); 4 Faculty of Veterinary Medicine, Ghent University, 9820 Merelbeke, Belgium; jeroen.dewulf@ugent.be; 5 FARAH Research Centre, Faculty of Veterinary Medicine, University of Liège, 4000 Liège, Belgium; a.linden@uliege.be (A.L.); claude.saegerman@uliege.be (C.S.); 6 Keiland, 9400 Ninove, Belgium; norbertsteurbaut@skynet.be; 7 ex FARAH Research Centre, Faculty of Veterinary Medicine, University of Liège, 4000 Liège, Belgium; etienne.thiry@uliege.be; 8 Animal Health Laboratory, Agence Nationale de Sécurité Sanitaire de L’alimentation, de L’environnement et du Travail, 94701 Maisons-Alfort Cedex, France; stephan.zientara@anses.fr

**Keywords:** bluetongue, virology, epidemiology, vector-borne disease, animal health, vaccination

## Abstract

Between 2006 and 2010, northwestern Europe experienced its first significant bluetongue virus (BTV) outbreak, driven by the spread of BTV-8, which had major repercussions on the European livestock sector. While BTV-3 was first identified in Europe in Italy in 2017, a new introduction of the virus was reported in 2023, in the Netherlands, and subsequently spread rapidly across the continent. A limited number of BTV-3 outbreaks were notified in Belgium in 2023, leading to the loss of its BTV-free status. In the following year, 2024, the virus spread throughout the country in a short time period. This study describes the impact of BTV-3 circulation in Belgium in 2024, detailing both its geographic spread and the associated increase in mortality, reduced births recorded, and decline in milk production among ruminants. Furthermore, preliminary results on the effectiveness of field vaccination and maternal immunity transfer are presented, as well as critical gaps that hinder the development of a robust, evidence-based management strategy. As the epidemiological situation is expected to become more complex in the future, due to the co-circulation of multiple BTV serotypes and other *Culicoides*-borne diseases, such as EHDV, effective collaboration and communication among stakeholders and international authorities will be crucial for implementing measures to mitigate the spread of these diseases.

## 1. Introduction

Bluetongue (BT) is a vector-borne viral disease transmitted by *Culicoides* midges. It is caused by bluetongue virus (BTV), a non-enveloped virus belonging to the *Orbivirus* genus within the family *Sedoreoviridae*, with a segmented genome consisting of 10 dsRNA segments encoding for seven structural proteins and at least four non-structural proteins [1]. As a result of the high variability of VP2, the structural protein encoded by segment 2, many BTV serotypes have been described [2]. Of these, 24 are classified as ‘typical serotypes’ and are notifiable under the Animal Health Law. Serotypes 1 to 24 are transmitted by midges. This does not seem to be the case for the ‘atypical serotypes’ 25 to 36, which have been shown to spread via direct contact between animals [3,4,5]. Moreover, the segmented genome organization of this virus leads to genomic segment reassortment possibilities which can play a role in virus evolution [6].

After a few sporadic incursions, BTV started to circulate in Europe between 1998 and 2005 with the detection of at least 5 serotypes (BTV 1, 2, 4, 9 and 16). Initially, the virus spread remained limited to the Mediterranean Basin. A first BTV-8 epidemic swept across northwestern Europe between 2006 and 2010, first reported in the Netherlands before spreading to Belgium, Germany, France and Luxembourg [7]. Between 2010 and 2023, different BTV outbreaks were notified in northern Europe associated with BTV-8 re-emergence as well as incursions of other BTV serotypes (e.g., BTV-4). Currently, a new BTV epidemic is spreading across Europe, this time linked to BTV-3. Although BTV-3 was first identified in Europe in Italy in 2017 [8], a new incursion was detected in the Netherlands in 2023 [9,10].

The disease has a seasonal distribution depending on the abundance of active vector populations, which is mediated through meteorologic conditions. In northwestern Europe, virus circulation and associated disease mainly occur from late spring to mid-autumn, corresponding to the period with an estimated optimum temperature of 20–21 °C for the activity of *Culicoides* from the Obsoletus group [11,12].

Many ruminant species are susceptible to BTV infection, but clinical presentation is species and strain/serotype-specific. Sheep are usually more severely affected than other domestic ruminants such as cattle or goats [13]. Clinical manifestations in sheep during the recent BTV-3 outbreak included hyperemia, apathy, hypersalivation and nasal discharge, erosions and ulcerations of the oral and nasal mucous membranes, facial edema, lesions of the coronary bands, lameness, and death [10,14]. This BTV-3 strain was also reported to induce BT-like clinical signs in cattle, and to a lesser extent in goats, ranging from subclinical and mild disease to severe manifestations, including death [14].

In addition to clinical disease, BTV circulation can also have a major economic impact on herds: first, through reduced animal production (e.g., milk yield and weight gain) and effects on reproduction (e.g., reduced fertility, abortion, congenital malformations); second, through international trade restrictions [9,15,16,17]. Although limited specific data are currently available on BTV-3, some impacts on production performances have already been reported based on increased mortality rates and drops in milk production, as well as suspected reduced reproductive performance [14,18,19].

BTV-3 control in Europe is further complicated due to the concomitant circulation of other BTV serotypes and Epizootic Hemorrhagic Disease virus serotype 8 (EHDV-8), another *Culicoides*-borne virus belonging to the same genus. All of these are notifiable diseases, yet it is impossible to distinguish them only based on clinical manifestation. In France, the concomitant presence of EHDV and BTV-3, 4 (Corsica) and 8 is currently complicating their control, while the detection of a BTV-12 introduction in the Netherlands was delayed due to the concomitant circulation of BTV-3 [20,21].

After its emergence in the Netherlands, a limited number of BTV-3 cases were also reported in Belgium in autumn of 2023, leading to the loss of its BTV-free status, although the extent of spread remained very limited. This was different in 2024, when a BTV-3 epidemic spread throughout the country from July onwards. The aim of this study is to describe the spread of the disease in Belgium over time in 2024, to evaluate its impact on production performances and to identify the current knowledge gaps hindering a scientifically underscored control program.

## 2. Materials and Methods

### 2.1. Confirmatory BTV-3 Diagnostics in 2024

Since BTV is a notifiable disease in Belgium, any clinical suspicion must be reported to the competent authority, and it is mandatory for veterinarians to collect and send blood samples from suspected animals (maximum three per farm) with clinical signs of BTV to the Belgian National Reference Laboratory (NRL, Sciensano). Upon receipt of the samples, real-time RT-PCRs are carried out to test for the presence of BTV [22] and confirm the serotype [23]. Negative samples for bluetongue were further tested for the presence of EHDV [24]. Based on the epidemiological situation with confirmed cases all over the country, the competent authority decided to suspend the mandatory RT-PCR testing of suspected cases from mid-September till the end of 2024.

### 2.2. Additional Samples for Virological and Serological BTV Testing

Besides the confirmatory testing, the NRL collaborated with a private veterinarian to collect samples from sheep belonging to five flocks from the province of East Flanders to preliminarily assess the immune response induced by vaccination and the transfer of maternal immunity. Information on the vaccination history, clinical signs, and lambing were provided by the veterinarian. If positive results for suspected samples were obtained, these were reported to the competent authority (see Section 2.1).

### 2.3. RNA Extraction and RT-qPCRs

RNA was extracted from EDTA blood samples using the Indimag 48 extraction robot with the Indimag Pathogen Kit (INDICAL BIOSCIENCE GmbH, Leipzig, Germany), following the manufacturer’s protocols.

A duplex RT-qPCR assay was used, combining a pan-BTV reaction targeting segment 10 encoding for NS3 [22] with an endogenous control reaction targeting GADPH, using the following primers and probe: GADPH_F (5′-TCACCATCTTCCAGGAGCGAG-3′), GADPH_R (5′-AAGGTGCAGAGATGATGACCCTC-3′) and GADPH_P (HEX-CAAGTGGGGTGATGCTGGTGCTGAGTA-BHQ1).

Amplifications were done using the AgPath-ID One-Step RT-PCR Kit (Thermo Fisher, Merelbeke, Belgium), with the following reaction mixture: 12.5 μL RT-PCR buffer (2×), 1 μL RT-PCR enzyme mix (25×), 2 μL mix primer/probe mix, 4.5 μL nuclease-free water and 5 μL denatured RNA extract. Final concentrations of primers and probes were 0.4 μM and 0.14 μM, respectively, for BTV, and 0.1 μM and 0.04 μM for GADPH. RNA denaturation was done by heating the RNA extracts at 95 °C for 3 min just before addition to the reaction mixture. Amplification was performed using the following cycling conditions:

45 °C for 10 min, 95 °C for 10 min, followed by 45 cycles at 95 °C for 15 s and 56 °C for 45 s. Samples with Ct-values ≤ 40 and showing exponential amplification curves were considered positive. A Ct value < 35 for the endogenous control (GAPDH) was required to consider a sample to be valid.

For the BTV-3-specific RT-qPCR targeting segment 2 encoding for VP2, primers and a probe, as described by Lorusso et al. (2018), were used [23]. The same amplification mix as for the pan-BTV assay was applied, with a modified annealing temperature of 60 °C instead of 56 °C. The final concentrations for BTV-3 primers were 0.8 µM, and for the probe, 0.32 µM.

A duplex RT-qPCR assay was also used for EHDV detection, combining a pan-EHDV reaction targeting segment 9, encoding for VP6 [24], with the same GAPDH endogenous control. Final concentrations of primers and probes were 0.4 μM and 0.2 μM, respectively, for EHDV, and 0.1 μM and 0.04 μM for GAPDH. The annealing temperature for this assay was 60 °C.

### 2.4. BTV ELISA

BTV antibodies were detected using a commercial VP7 competition enzyme-linked immunosorbent assay (ELISA) (ID SCREEN Bluetongue, Innovative Diagnostics, France) following the manufacturer’s instructions. Results were expressed as a competition percentage (S/N%), calculated using the formula (OD of the sample/OD of the negative control) × 100. Samples with S/N% values ≥ 40 were classified as negative and values < 40 as positive.

### 2.5. BTV VNT

The VNT was conducted to evaluate the presence of neutralizing antibodies against the BTV-3 strain BEL 2024/01. BTV-3-positive control antisera (NVRL BTV 03 PS 001) and negative bovine sera were included as positive and negative controls. Serum samples were decomplemented for 30 min at 56 °C. A quantity of 50 µL of two-fold serially diluted serum from 1:10 to 1:1280 was incubated with 50 μL of virus dilution containing 100 TCID_50_ of the BTV-3 strain for 1 h at 37 °C. After 1 h, 50 μL of BHK (Baby Hamster Kidney) cell suspension (2 × 10⁴ cells) was added per well. After a 3-day incubation period at 37 °C, wells were evaluated under the light microscope for the presence of a cytopathic effect (CPE). The neutralizing antibody titer was defined as the reciprocal of the highest serum dilution that neutralized CPE in 50% of the wells.

### 2.6. Geographical Mapping of Confirmed BTV Cases

The Veterinary Epidemiology Service of Sciensano produced maps updated daily and showing the number and location of BTV-confirmed herds.

Data (PCR results) were extracted from the Laboratory Information Management System (LIMS) of Sciensano, along with metadata related to tested animals (animal and herd ID, herd location, animal species, etc.). Herds with at least one PCR-positive animal were considered as confirmed BTV outbreaks. The number of outbreaks was aggregated at the municipality level, and each municipality was then categorized according to the number of recorded outbreaks since June 1st, marking the start of the vector activity period. Maps displaying the municipalities and their respective categories (0, 1, 2, 3–5, >5 outbreaks), visualized by different color codes, were generated daily and posted on the Sciensano website throughout the epidemic (https://www.sciensano.be/en/about-sciensano/sciensanos-organogram/exotic-and-vector-borne-diseases/epidemiologic-situation-bluetongue, accessed on 26 March 2025). Data analysis and mapping were performed using R, version 4.2.2.

### 2.7. Data on Ruminant Mortality, Births and Milk Production

The Belgian Veterinary Authorities have access to data on ruminant mortality, births and milk production. Data on cattle births are registered within the Identification and Registration system (SANITEL). Data on cattle mortality are available via the database of the only Belgian rendering facility responsible for the carcass collection activities in Belgium (RENDAC). Milk production data can be accessed from the sector federation of the Belgian dairy industry (BCZ/CBL), which represents over 99% of all milk volume collected in Belgium.

### 2.8. Calculation of Mortality Rates

The number of cattle and small ruminants in Belgium on the first of December of each year (2020–2024) was extracted from the Identification and Registration system (SANITEL).

The mortality rates were calculated by dividing the number of animal deaths by the total number of animals recorded on 1 December for each corresponding year. However, due to a difference in census methodology (continuous census for cattle versus census in December for small ruminants), the total number of animals recorded on the 1 December for each previous year was used for small ruminants.

### 2.9. Statistical Analysis

A Mann–Whitney test was performed to evaluate potential significant differences in the titers between the ewes and the lambs. Statistical analyses were performed in GraphPad Prism 9, and differences were considered significant if *p* < 0.05.

## 3. Results and Discussion

### 3.1. BTV-3 Spread in Belgium

**Figure 1:**
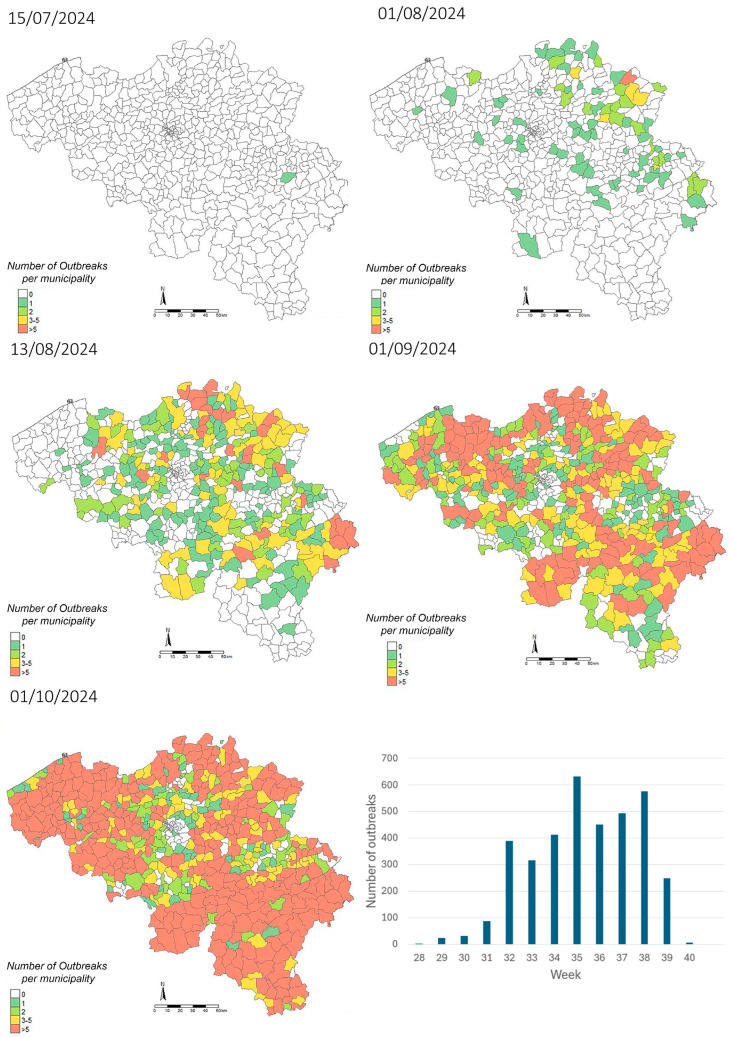
Evolution of the distribution of BTV-3 outbreaks reported in Belgium between 15 July 2024, and 1 October 2024 and evolution of the number of outbreaks recorded per week between week 28 (8 July 2024 to 14 July 2024) and week 40 (30 September 2024 to 6 October 2024), based on tests sent for clinical suspicion of BTV cases (Sciensano Institute, NRL for bluetongue disease, available at https://moriskin.sciensano.be/shiny/bluetongue/, accessed on 26 March 2025).

The first BTV-3 case of 2024 following the resumption of vector activity was confirmed on 9 July in cattle in Sprimont, in the province of Liège (Figure 1). The number of confirmed cases slowly increased in the following weeks, with 77 confirmed herds by the end of July. Starting from then, an epidemic spread took place, with BTV confirmed cases in all provinces by 2 August. By 16 September, the moment that the veterinary authorities decided to suspend confirmatory testing on suspected cases, BTV had been confirmed in 2903 herds, including 1824 outbreaks in cattle, 1021 in sheep, 39 in goats, 17 in alpacas and 2 in captive cervids. Due to the time gap related to sample reception and testing, these results are reflected in the map of 1 October. No other BTV serotype nor EHDV was detected in Belgium in 2024, given that all BTV-positive samples were also confirmed positive for BTV-3 and all BTV-negative samples were confirmed negative for EHDV. While no additional serotype-specific tests were performed on the BTV-3-positive samples, the Ct values obtained with the pan-BTV assay were highly comparable to those from the BTV-3-specific assay, suggesting that BTV-3 is the probable source of the pan-BTV-positive results. Nevertheless, the presence of co-infections with other BTV serotypes cannot be formally excluded.

The observed rapid spread of the disease over the whole territory was not surprising. Similar epidemic spread had been reported during the BTV-8 outbreak in Europe in 2006 [7] and in 2023 for the outbreak of BTV-3 in the Netherlands [14]. An average disease propagation rate of 5.6 km/day has previously been estimated for BTV-8 via natural dispersal of infected vectors [25]. A number of factors certainly contributed to the rapid spread of BTV-3 in Belgium in 2024, namely the presence of competent *Culicoides* species, favorable climatic conditions, and the presence of an almost completely naive population. In 2023, only a few clinically diseased sheep were reported in late autumn, which were confirmed BTV-3-positive by RT-PCR. A retrospective serological study in Belgian sheep and cattle herds carried out in January and February 2024 confirmed that virus circulation had remained very limited. Additional contributing factors included the absence of cross-protection from exposure or vaccination against other serotypes such as BTV-8 and the late availability of BTV-3 vaccines for the 2024 vector season [12,26,27]. Indeed, three inactivated vaccines against BTV-3 from three different companies (Syvazul BTV-3, Syva S.A.; BULTAVO 3, Boehringer Ingelheim; and BLUEVAC-3, CZ Vaccines S.A.U.) were available in Belgium in the late spring of 2024 and were used to vaccinate cattle and sheep on a voluntary basis. As these vaccines had no complete market authorization but only an authorization to use in emergency, as allowed by Regulation (EU) 2019/6 (article 110), data about safety, efficacy, duration of immunity, etc. provided by the manufacturers were limited, hindering a well-defined vaccination strategy and underlining the importance of implementing effective communication with the sector.

### 3.2. Impact of BTV-3 Circulation on Livestock Production

The BTV-3 circulation in 2024 had an important impact on ruminant farming in Belgium. The direct effect on mortality, reproduction and milk production can be readily quantified based on available production data and is discussed below in more detail.

#### 3.2.1. Excess Mortality in Cattle and Small Ruminants

**Figure 2:**
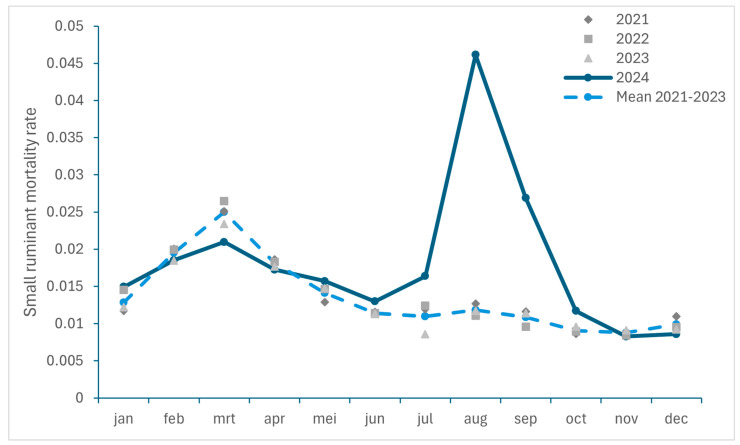
Monthly small ruminant mortality rate recorded in Belgium between 2021 and 2024 and monthly mortality mean for 2021 to 2023.

To get an insight in the excess mortality caused by BTV-3 infections in 2024, the ratios between the monthly number of dead ruminants collected by RENDAC during the period 2021–2023 and 2024 and the total number of animals on 1st December of each year were compared in Figure 2 (for small ruminants) and 3 (for cattle). It should be noticed that a decrease in the number of cattle (−93,797 animals) as well as in the number of cattle herds (−587) was recorded between January and December 2024. As we considered the number of animals recorded on 1 December 2024 for the calculation of the mortality ratio, this could lead to an overestimation. However, as the number of cattle deaths was extracted from the rendering facility responsible for the carcass collection and does not include animals sent for slaughter, it can be assumed that the calculated ratio for mortality should be representative of the situation in 2024.

An increase in mortality was observed in both cattle and small ruminants with the highest excess in mortality recorded in August and September 2024, with an increase of 46% and 54% for cattle, and of 290% and 148% for small ruminants, respectively.

Over the period from July to December, the excess mortality among small ruminants was 92% (corresponding to an additional 24,519 dead small ruminants (sheep and goats); approximately 6% of the Belgian small ruminant population in 2024) and higher than the 30% excess mortality observed in cattle (equal to an additional 21,142 dead cattle; approximately 1% of the Belgian cattle population in 2024).

**Figure 3:**
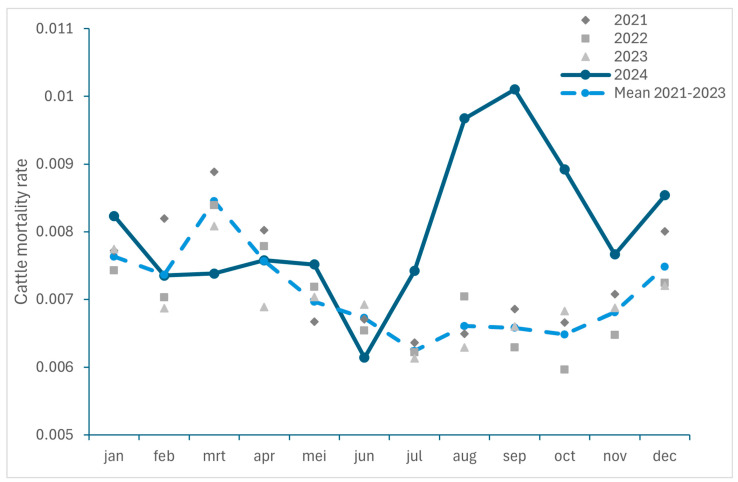
Monthly cattle mortality rate recorded in Belgium between 2021 and 2024 and monthly recorded mortality mean for 2021 to 2023.

The number of rendered ruminants in November 2024 returned to levels comparable to those in 2023, with even a slight decrease in mortality (−13%; Figure 3) recorded in December for small ruminants.

Differences in the recorded number of deaths animals per cattle category were observed, with a higher excess mortality recorded for calves up to 25 kg and up to 10 kg, as well as for bovines of 800 kg, while other cattle categories were less impacted (see Table S1). Moreover, there was still a fairly high excess of recorded deaths among calves up to 10 kg and up to 25 kg in November and December (see Table S1).

This observed increase in ruminant mortality is consistent with the findings in the Netherlands with regard to the BTV-3 outbreak in 2023. Indeed, a significant increase in mortality was described in small ruminants, especially in sheep, with an additional 55,000 dead sheep (approximately 4.5% of the Netherlands’ sheep population) during the BTV-3 outbreak period in 2023 compared to the same period in 2020–2022 [18]. Moreover, an increase in mortality was also registered in cattle herds during this period when comparing infected herds, herds located in BTV-3-infected areas and herds in BTV-3-free areas with a higher mortality recorded in young stock (1–2 years) and cattle ≥ 2 years of age [19]. The previous BTV-8 epidemic has also been associated with increased mortality in cattle and sheep [17,28,29,30]. However, the increase in mortality associated with BTV-3 could be higher, with a case fatality rate in some herds of 74.8% described for sheep [14] compared with case fatality rates of between 37.5% and 50% reported for sheep during the BTV-8 epidemic in the Netherlands and Germany and between 6.4 and 13.1% for cattle [29,31].

The observation that the excess mortality almost disappeared in November is likely linked to the end of the vector period around this time and the associated drop in virus circulation. Yet this cannot be confirmed with certainty, as no *Culicoides* monitoring is in place in Belgium.

#### 3.2.2. Decrease in Number of Newborn Calves

**Figure 4:**
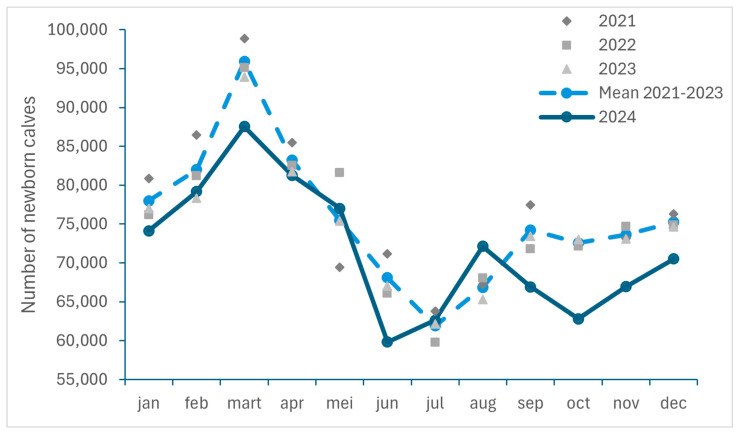
Monthly number of newborn calves registered in Belgium between 2021 and 2024 and monthly mean for the years 2021 to 2023 (SANITEL).

The monthly number of newborn calves in Belgium between 2021 and 2024, as well as the monthly mean for the years 2021 to 2023, are presented in Figure 4. This number was consecutively lower in September to December 2024 than the mean number for those months in the period 2021–2023, with an observed decrease of respectively 9.85%, 13.43%, 9.09%, 6.31%.

This drop in the number of births in parallel with the circulation of BTV-3 could be explained by the effect of the virus on fetuses in the event of transplacental infection, such as abortions, congenital malformations or nervous clinical signs in newborn calves, as described during the BTV-8 epidemic [15,32,33]. The hypothesis of a transplacental infection of BTV-3 with comparable repercussions was raised during the 2024 epidemic. It is supported by an increase in the number of declared abortions in Belgium in 2024, as well as the observation of congenital malformations which can be associated with *Orbivirus* infections in some autopsied fetuses. These findings are consistent with the increase in the number of abortions, premature births and malformations recorded in Belgium during the BTV-8 epidemic [17,34]. Based on a study evaluating the impact of BTV-3 on cattle mortality, abortions and premature births in the Netherlands in 2023, the rate of abortions was also higher in infected herds than in the other herds, but no significant association between BTV-3 infections and abortions (between 180 days and 260 days of gestation) was found [19]. These results are in accordance with another study describing no clear effect of BTV-8 infection on abortion rates in the Netherlands in 2008 [35]. However, an increase in perinatal calf mortality (including late abortions, stillbirths and calves that died within 3 days after birth (timeframe before mandatory identification) was described, as well as a higher percentage of premature births (between 260 and 265 days gestation) in BTV-3-infected herds than in BTV-3-free herds [19].

If BTV-3 infections are similar to BTV-8 infections, a reduced number of calves born in the first months of 2025 can also be expected. BTV-8 infection was shown to impact male fertility, on the one hand, by reducing sperm motility and increasing the percentage of spermatozoa with morphological abnormalities [36,37,38] and female fertility, on the other hand, with an increased return-to-service period as well as more inseminations needed for an assumed pregnancy, inducing a higher interval between calving [17,35]. The effect of infection during gestation can vary depending on the potential effects of the virus on the different stages of conception and gestation, going from conception failure, embryonic death and fetal death in early gestation to abortions or congenital malformations in later stages [35,39,40] but probably also on the female reproduction organs as ovaries.

#### 3.2.3. Impact on Milk Deliveries

**Figure 5:**
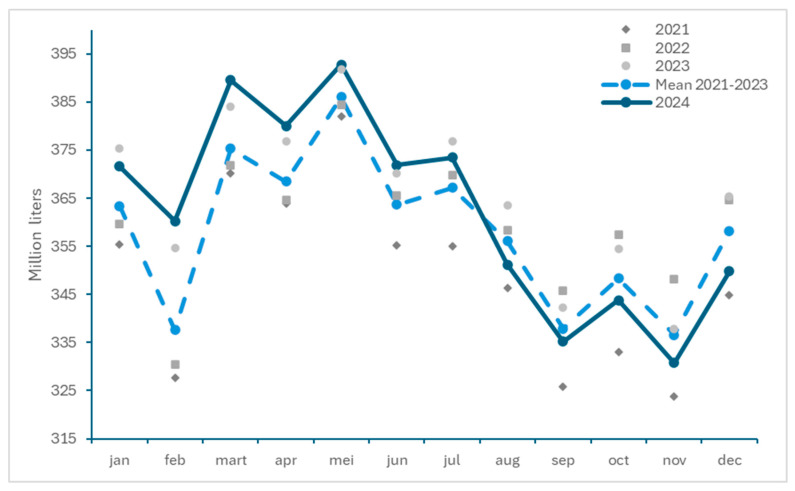
Monthly milk deliveries in Belgium between 2021 and 2024 and mean for the years 2021 to 2023 (from BCZ/CBL, https://bcz-cbl.be/sites/default/files/2025-02/Melkleveringen_12_NL.pdf; https://bcz-cbl.be/sites/default/files/2023-10/melkeveringen_2021_nl.pdf, accessed on 26 March 2025).

The monthly milk deliveries in Belgium between 2021 and 2024 are presented in Figure 5. Although higher milk volumes were delivered in the first half of 2024 compared to previous years, a drop in milk deliveries was observed concomitant with the period of BTV-3 circulation. Decreases of 1.36%, 0.80%, 1.29%, 1.72% and 2.37% in the months August to December 2024 compared to the mean of 2021–2023, respectively, were reported [41]. The available data, however, show that differences exist in milk deliveries between the years, with, e.g., lower milk deliveries in July to December 2021 than in the corresponding months in 2024, while this was opposite in 2022 and 2023.

This observed reduction in milk deliveries in 2024 compared to the mean of the previous 3 years are comparable with those reported for the Netherlands in the same period, where they respectively fell by 3.47%, 2.61%, 2.08%, 1.84% and 1.31% [42]. Due to the increased milk deliveries in the first half of 2024, a global increase in milk production of 1.20% was registered in Belgium for the year 2024, which was different compared to the Netherlands, which reported an overall decrease of −1.15% for 2024.

In contrast to Belgium, where a drop in milk production became noticeable from August, the Netherlands observed a reduction in milk deliveries for every month except February. This earlier drop in milk production is probably linked to the fact that BTV-3 had already extensively circulated in 2023, and restarted circulating earlier in 2024 in the Netherlands than in Belgium.

The provided information on reduced milk deliveries is representative for the overall situation, but may mask particular cases (farms or regions) in which the drop in milk deliveries may have been even more important. Moreover, and in line with the observations in the Netherlands in 2024, this effect is likely to become more pronounced in 2025 in Belgium as the calving season progresses, following a potential accumulation of abortions, and associated reduced milk production.

#### 3.2.4. Conclusions

Based on data relating to direct production losses associated with the reduction in births, the increase in mortality and the drop in milk production and their temporal association with the BTV-3 epidemic, it can be concluded that the circulation of BTV-3 did have an impact on ruminant farming in Belgium in 2024. However, all these parameters are multifactorial, and it cannot be ruled out that other elements, such as, for example, seasonal variation, decreased feed quality or quantity, other diseases circulation, etc., may also have had an impact on the evolution of these data [43].

Besides these direct production losses which can have an economic impact on the sector, indirect additional costs can also occur, such as trade restrictions for animals and animal products (semen), disease surveillance (diagnostics) or disease control and prevention (vaccination, indoor housing, insecticide treatment). These costs are difficult to estimate for the epidemic of BTV-3 in Belgium in 2024 due to lack of data. However, direct and indirect costs of previous BTV epidemics have been evaluated in the literature. In the Netherlands, the overall cost of the BTV-8 epidemic was estimated to be, respectively, EUR 32.4 million in 2006 and EUR 164–175 million in 2007. In 2006, 98% of these costs were due to indirect costs (control measures and diagnostics), while 92% were due to direct costs (production losses and veterinary treatment fees) in 2007. This can be linked to the fact that control measures were less stringent during the second year of the epidemic and to the huge spread of the virus, which was associated with an important increase in the number of affected farms [44]. In Germany, the impact of the BTV-8 epidemic (from the first cases in 2006 until 2018) ranged between EUR 157 and 203 million, of which 73% was due to indirect costs (vaccination, insecticide treatment, diagnostic testing for trade, monitoring and surveillance, administration) and 27% to direct costs [15]. Considerable costs were also estimated in Belgium (Wallonia) for the 2006–2007 epidemic with an estimated cost of EUR 205 per head of beef cattle, EUR 233 per head of dairy cattle and EUR 53 per sheep in infected herds and flocks, mainly due to reproductive disorders, drops in milk production, mortality and veterinary costs [17]. However, these figures must be interpreted with caution, as the various studies do not take the same parameters nor the same years of virus circulation into account in their cost assessments. Moreover, the financial consequences may also differ depending on the sector and type of production (e.g., dairy or beef animals). In conclusion, further effects of BTV-3 circulation should still be observed in 2025, and it is still too early to estimate the total losses generated due to BTV-3 circulation in Belgium. It would be interesting to carry out a quantified assessment of the economic impact of BTV-3 epidemics in 2024 when all the data are available.

### 3.3. Research Recommendations Based on Identified Knowledge Gaps

#### 3.3.1. Additional Aspects on Vaccine Protection from BTV-3 Vaccination in the Field

**Figure 6:**
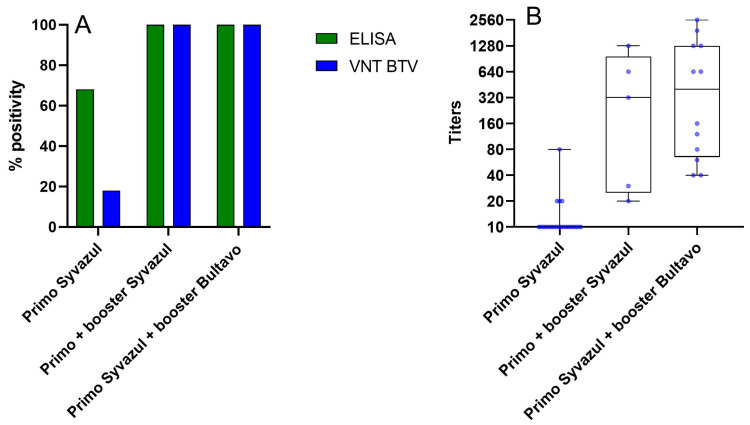
ELISA (**A**) and VNT (**B**) results after sheep primo vaccination with Syvazul, primo and booster Syvazul and primo Syvazul and booster Bultavo.

To get some preliminary information about vaccine efficacy under field conditions, a small-scale field assay was performed in collaboration between Sciensano and a private veterinary practice. Twenty-two sheep were sampled between 20 and 26 July, before BTV-3 was detected in East Flanders, approximately two months after receiving a primo vaccination with Syvazul BTV-3. Only 68% of these sheep tested positive in the BTV ELISA and only 18% tested positive in the VNT BTV-3, with very low titers (Figure 6). These preliminary data suggest that a booster vaccination is essential to achieve sufficient immunity against BTV-3 infection, as the neutralizing antibody response following the primo vaccination remains low. This observation is also in line with reported breakthrough infections after primo vaccination from the field in Belgium and in the Netherlands.

On the day of sampling, 5 sheep received a booster vaccination with Syvazul BTV-3, while 12 sheep received a booster vaccination with BULTAVO 3. Serum samples were collected again at 28 days post-booster for Syvazul BTV-3 (23 August 2024) and 21 days post-booster for BULTAVO 3 (11 and 12 August 2024). At this time point, all tested samples were positive in both ELISA and VNT BTV-3. In the ELISA assay, all samples exhibited a strong response, whereas the VNT results showed a broad range of titers, with some animals reaching high antibody levels (see Figure 6). Whether these high titers were the sole result of the booster vaccination or due to an additional natural infection cannot be ascertained, as these samples were collected in August 2024, when BTV-3 was already circulating in East Flanders. Two animals with low VNT titers (1/30 and 1/20) after booster vaccination with Syvazul showed clinical signs compatible with BTV-3 infection and were confirmed BTV-3-positive by RT-PCR in blood samples.

It has been shown for BTV-8 that vaccination of pregnant animals was advantageous, as it prevented transplacental transmission, which was linked to abortions or congenital malformations following BTV-8 infection. On the other hand, transferred maternal immunity interfered with vaccination of young animals [15,45,46]. Various studies on BTV-8 evaluated the interference of maternal antibodies in the response to BTV vaccination, and it was shown that lambs from hyperimmune ewes (naturally infected with BTV-8 in 2007 and vaccinated several times thereafter) vaccinated at 3 months of age may not respond optimally to vaccination, while vaccination at 5, 7 and 9 months of age protects lambs both clinically and virologically [47]. In addition, vaccination of calves from BTV-8 vaccinated cows at 7 months of age was shown to result in a good level of protection [45].

**Figure 7:**
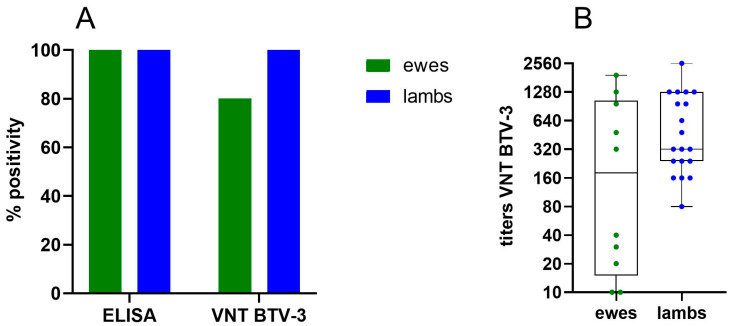
ELISA (**A**) and VNT (**B**) results of ewes and lambs.

To get a preliminary idea of transfer of maternal immunity against BTV-3, serum samples were collected from 10 ewes, which were primo and booster vaccinated with Syvazul BTV-3 in 2024, and their 19 lambs shortly after birth (+/−10 days) in January 2025. ELISA results showed strong positivity in all ewes and lambs. In the VNT, 8 out of 10 ewes tested positive, while all 19 lambs had neutralizing antibodies (Figure 7A). As illustrated in the graph (Figure 7B), VNT titers varied widely. High titers are likely associated with the infection of some ewes during the summer of 2024, while lower titers are probably related to vaccination. However, statistical analysis using the Mann–Whitney test revealed no significant difference in titers between ewes and lambs (*p* = 0.19). Remarkably, neutralizing antibodies were detected in the lambs born from the VNT-negative ewes. One possible explanation is that maternal antibodies were present in the ewes at insufficient levels to be detected in the blood but were concentrated in the colostrum and transferred to the lambs. In sheep, immunoglobulin G (IgG) is transported from the bloodstream to the mammary gland before birth, accumulating in the colostrum while decreasing in the ewe’s blood to provide passive immunity to newborns [48]. Another possibility is that some lambs consumed colostrum from other ewes in the group.

These preliminary data indicate that maternal antibodies against BTV-3 are transferred from ewes to lambs through colostrum intake. The continued monitoring of the lambs to determine how long the maternal antibodies remain in their circulation will be crucial for determining the optimal timing for vaccinating the lambs in 2025 and the following years.

As a response to the extensive spread of BTV-3 and associated production losses in 2024, Belgium decided to implement a mandatory BTV-3 and BTV-8 vaccination in 2025. This should achieve a sufficient vaccination coverage of the ruminant population, which is necessary to contain a vector-borne disease such as BTV [49]. However, the limited efficacy data, the absence of information about the duration of immunity, the transfer of maternal immunity, and the interference of maternal antibodies with vaccination of lambs born from vaccinated ewes complicate the elaboration of an effective evidence-based vaccination strategy. In this context, the results of the ongoing vaccination studies will need to be translated into vaccination advisories in real time to ensure that the compulsory vaccination campaign will be successful.

#### 3.3.2. BTV-3 Transmission Through Sperm and Embryos

Data supporting the risk of BTV-8 transmission via semen or embryo transfer are available in the literature. BTV-8 has been detected both by PCR and by virus isolation in semen of naturally infected animals, and transmission of BTV-8 following artificial insemination with contaminated frozen semen has also been described [50,51,52]. Additionally, although the risk of BTV transmission during embryo transfer is considered negligible for different serotypes when proper washing procedures are applied [53,54], transmission of BTV-8 to in vitro exposed embryos has been reported despite the implementation of correct washing protocols. This highlighted the importance of screening potential donors and collected embryos [54]. Again, few specific data are currently available for BTV-3, considering that the risk of transmission varies between the studied serotype in the literature. Available information indicates that this risk is higher for BTV-8 (and viruses adapted to laboratory cell culture) than for other serotypes.

During the summer of 2024, a significant number of bulls from artificial insemination centers were infected with BTV-3. This is a major concern, as European regulations (Regulation (EU) 2020/686 and Directive 92/65/EEC) prohibit the use of semen from BTV-positive bulls for artificial insemination. The problem is further complicated by the prolonged RT-PCR-positive status of infected bulls. Findings from the Belgian NRL confirm blood samples from several bulls still being BTV RT-PCR-positive in January 2025, approximately six months after the initial infection.

Studies evaluating the potential transmission of BTV-3 through the use of germinal products and assessing the duration for which these remain infectious are needed in order to establish a risk-based management.

#### 3.3.3. Diagnostic Challenges

BTV-8 has, for a long time, been the only serotype present in Belgium. However, the recent introduction of BTV-3 and the presence of other serotypes (e.g., BTV-8 in France, BTV-12 in the Netherlands) or related viruses (e.g., EHDV-8 in France) in neighboring countries poses a challenge for effective diagnostic strategies, as these viruses induce similar clinical manifestations.

The routine diagnostic strategy in case of a suspicion currently applied by the NRL consists of pan-BTV RT-PCR testing, followed by serotyping in case of a positive result. However, due to the co-circulation of multiple viruses inducing similar symptoms, there is a need for cost-effective alternative diagnostic approaches. A possible solution could be the implementation of multiplex RT-PCRs, which would allow the direct differentiation between various BTV serotypes or different viruses (BTV/EHDV). It remains, however, to be decided based on the epidemiological situation which viruses should be included in such a multiplex, and to be evaluated what the impact of multiplexing on test sensitivity and specificity would be, to ensure that no diagnostic performance is lost compared to the currently validated single-plex RT-PCR test.

While PCR-based methods are crucial for confirmatory diagnostics, serological tests are valuable tools for monitoring virus circulation and herd-level exposure over time. Commercial ELISAs are currently most often used for detection of BTV-specific antibodies in serum. These tests are easy to perform and provide quick results, but they cannot distinguish between infection with the different serotypes, nor determine whether the detected antibodies are the result of infection or vaccination of the animals. This method is, therefore, mostly useful to monitor the circulation of the virus in previously free regions, but becomes less informative for surveillance in regions where vaccination and active BTV circulation may co-occur. In addition, the aspect of cross-reactivity between different orbiviruses in ELISA needs to be considered. The Belgian NRL has observed cross-reactivity of strong BTV-3-positive sera in a commercial EHDV ELISA, which could further complicate the interpretation of ELISA results in the event of co-circulation of both BTV and EHDV in the future.

Besides serum testing, ELISA can also be used for milk samples. A specific ELISA kit is available for BTV antibody detections in milk, which is also suitable for bulk tank milk screening. This method allows cost-effective herd-level surveillance but has limitations, including lower sensitivity in low-prevalence herds and the inability to identify individual seropositive animals.

The virus neutralization test (VNT) provides an alternative to ELISAs by detecting neutralizing antibodies. VNTs allow researchers to determine to which virus and serotype an animal has been exposed by vaccination or infection. However, they require working with infectious virus and are labor intensive and costly, and therefore not suitable for large-scale testing.

Finally, determining the whole genome sequence of BTV strains present in selected samples might be important, certainly when multiple serotypes co-circulate. This allows researchers to detect reassortment events that may occur when different strains circulate in the same area and provide information to ensure that vaccines should still be effective and to adapt the control measures accordingly.

#### 3.3.4. Role of Wildlife in BTV-3 Epidemiology

Many ruminant species are susceptible to BTV infection, and it is suspected that wild ruminants may play a role as reservoir and thus contribute to maintaining disease endemicity.

According to a study carried out between 2005 and 2008 in Belgium, BTV-8 circulated in free-ranging cervids, with a recorded seroprevalence of above 50% in red deer (*Cervus elaphus*). These, nevertheless, had no gross lesions compatible with bluetongue disease [55]. A decrease in seroprevalence was observed in 2008 and might have been caused by a reduction in virus circulation associated with the mandatory vaccination of captive ruminants as well as to increased herd immunity within the red deer population. Data on BTV-3 presence or circulation in wildlife are currently lacking. As control measures may be impacted by a wildlife reservoir in which the virus could circulate, both the prevalence of BTV-3 in the wildlife and its reservoir role should be clarified and monitored.

In addition, captive wild ruminants can also be infected with BTV [56,57,58] and the presence of BTV-3 has been confirmed by RT-PCR in blood samples from suspected captive European bison (*Bison bonasus*), American bison (*Bison bison*) and gazelle (*Nanger dama mhorr)* from a Belgian zoo (Unpublished data). The extent of BTV-3 infection in captive wildlife should be studied in more detail, and it should be considered to vaccinate susceptible wild species in zoos, as these gather large numbers of valuable animals whose clinical infection could result in substantial losses.

## 4. Conclusions

The rapid spread of BTV-3 across Belgium in 2024 was associated with direct production losses impacting the livestock industry, although overall quantitative economic losses due to the BTV-3 outbreak remain to be determined. Several knowledge gaps still remain about BTV-3 epidemiology, virus-host interactions and vaccine-related parameters, and efforts should be undertaken to study these knowledge gaps in order to refine the current disease control strategy.

The epidemiological situation in Europe becomes increasingly complex due to the circulation of several BTV serotypes and other orbiviruses like EHDV. Early detection by strong passive surveillance combined with an appropriate diagnostic testing strategy therefore becomes crucial, together with the availability of effective vaccines to allow rapid disease control. In this context, it is essential to implement good collaboration and communication between the various stakeholders and international authorities in order to take necessary measures to limit the spread and impact caused by these diseases.

## Data Availability

All available data are in the manuscript.

## Supplementary Materials

The following supporting information can be downloaded at: https://www.mdpi.com/article/10.3390/v17040521/s1, Table S1: Excess number of dead animals recorded in 2024 compared to the mean of the years 2021 to 2023 (%) in the different cattle categories.

## Abbreviations

The following abbreviations are used in this manuscript:

BT	Bluetongue
BTV	Bluetongue Virus
EHDV	Epizootic Hemorrhagic Disease Virus
IgG	Immunoglobulin G
NRL	National Reference Laboratory
PCR	Polymerase Chain Reaction
VNT	Virus Neutralization test

## References

[1] Rodríguez-Martín D., Louloudes-Lázaro A., Avia M., Martín V., Rojas J.M., Sevilla N. (2021). The Interplay between Bluetongue Virus Infections and Adaptive Immunity. Viruses.

[2] WOAH Terrestrial Manual 2021. Chapter 3.1.3. Bluetongue (Infection with Bluetongue Virus). https://www.woah.org/fileadmin/Home/fr/Health_standards/tahm/3.01.03_BLUETONGUE.pdf.

[3] Belbis G., Zientara S., Bréard E., Sailleau C., Caignard G., Vitour D., Attoui H. (2017). Bluetongue Virus: From BTV-1 to BTV-27. Adv. Virus Res..

[4] Ries C., Vögtlin A., Hüssy D., Jandt T., Gobet H., Hilbe M., Burgener C., Schweizer L., Häfliger-Speiser S., Beer M. (2021). Putative Novel Atypical BTV Serotype ‘36’ Identified in Small Ruminants in Switzerland. Viruses.

[5] Spedicato M., Compagni E.D., Caporale M., Teodori L., Leone A., Ancora M., Mangone I., Perletta F., Portanti O., Di Giallonardo F. (2022). Reemergence of an Atypical Bluetongue Virus Strain in Goats, Sardinia, Italy. Res. Vet. Sci..

[6] Nomikou K., Hughes J., Wash R., Kellam P., Breard E., Zientara S., Palmarini M., Biek R., Mertens P. (2015). Widespread Reassortment Shapes the Evolution and Epidemiology of Bluetongue Virus Following European Invasion. PLoS Pathog..

[7] Saegerman C., Berkvens D., Mellor P.S. (2008). Bluetongue Epidemiology in the European Union. Emerg. Infect. Dis..

[8] Lorusso A., Guercio A., Purpari G., Cammà C., Calistri P., D’Alterio N., Hammami S., Sghaier S., Savini G. (2017). Bluetongue virus serotype 3 in Western Sicily, November 2017. Vet. Ital..

[9] Niedbalski W. (2022). Bluetongue Virus in Europe: The Current Epidemiological Situation. Med. Weter..

[10] Holwerda M., Santman-Berends I.M.G.A., Harders F., Engelsma M., Vloet R.P.M., Dijkstra E., van Gennip R.G.P., Mars M.H., Spierenburg M., Roos L. (2024). Emergence of Bluetongue Virus Serotype 3 in the Netherlands in September 2023. Emerg. Infect. Dis..

[11] Möhlmann T.W.R., Keeling M.J., Wennergren U., Favia G., Santman-Berends I., Takken W., Koenraadt C.J.M., Brand S.P.C. (2021). Biting Midge Dynamics and Bluetongue Transmission: A Multiscale Model Linking Catch Data with Climate and Disease Outbreaks. Sci. Rep..

[12] European Centre for Disease Prevention and Control (ECDC) Biting Midge Maps. https://www.ecdc.europa.eu/en/disease-vectors/surveillance-and-disease-data/biting-midge-maps.

[13] Guyot H., Mauroy A., Kirschvink N., Rolin F., Saegerman C., Saegerman C., Reviriego-Gorrdejo F., Pastoret P.P. (2008). Clinical Aspects of Bluetongue in Ruminants. Bluetongue in Northern Europe.

[14] van den Brink K.M.J.A., Santman-Berends I.M.G.A., Harkema L., Scherpenzeel C.G.M., Dijkstra E., Bisschop P.I.H., Peterson K., van de Burgwal N.S., Waldeck H.W.F., Dijkstra T. (2024). Bluetongue Virus Serotype 3 in Ruminants in the Netherlands: Clinical Signs, Seroprevalence and Pathological Findings. Vet. Rec..

[15] Saegerman C., Bolkaerts B., Baricalla C., Raes M., Wiggers L., de Leeuw I., Vandenbussche F., Zimmer J.-Y., Haubruge E., Cassart D. (2011). The Impact of Naturally-Occurring, Trans-Placental Bluetongue Virus Serotype-8 Infection on Reproductive Performance in Sheep. Vet. J..

[16] Gethmann J., Probst C., Conraths F.J. (2020). Economic Impact of a Bluetongue Serotype 8 Epidemic in Germany. Front. Vet. Sci..

[17] Hanon J.-B., Uyttenhoef A., Fecher-Bourgeois F., Kirschvink N., Haubruge E., Duquesne B., Saegerman C. (2009). Estimation quantitative des pertes économiques directes et indirectes subies par les éleveurs wallons dans le cadre de la fièvre catarrhale ovine (sérotype 8) durant la période 2006–2007. Epidémiologie Santé Anim..

[18] Santman-Berends I.M.G.A., van den Brink K.M.J.A., Dijkstra E., van Schaik G., Spierenburg M.A.H., van den Brom R. (2024). The Impact of the Bluetongue Serotype 3 Outbreak on Sheep and Goat Mortality in the Netherlands in 2023. Prev. Vet. Med..

[19] van Den Brink K.M.J.A., Brouwer-Middelesch H., Van Schaik G., Lam T.J.G.M., Stegeman A., Van Den Brom R., Spierenburg M.A.H., Santman-Berends I.M.G.A. The Impact of Bluetongue Serotype 3 on Cattle Mortality, Abortions and Premature Births in the Netherlands in the First Year of the Epidemic. https://ssrn.com/abstract=5056290.

[20] Gondard M., Postic L., Garin E., Turpaud M., Vorimore F., Ngwa-Mbot D., Tran M.-L., Hoffmann B., Warembourg C., Savini G. (2024). Exceptional Bluetongue Virus (BTV) and Epizootic Hemorrhagic Disease Virus (EHDV) Circulation in France in 2023. Virus Res..

[21] van den Brom R., Santman-Berends I., van der Heijden M.G., Harders F., Engelsma M., van Gennip R.G.P., Maris-Veldhuis M.A., Feddema A.-J., Peterson K., Golender N. (2025). Bluetongue Virus Serotype 12 in Sheep and Cattle in the Netherlands in 2024–A BTV Serotype Reported in Europe for the First Time. Vet. Microbiol..

[22] Hofmann M., Griot C., Chaignat V., Perler L., Thür B. (2008). Blauzungenkrankheit Erreicht Die Schweiz. Schweiz. Arch. Tierheilkd..

[23] Lorusso A., Sghaier S., Di Domenico M., Barbria M.E., Zaccaria G., Megdich A., Portanti O., Ben Seliman I., Spedicato M., Pizzurro F. (2018). Analysis of Bluetongue Serotype 3 Spread in Tunisia and Discovery of a Novel Strain Related to the Bluetongue Virus Isolated from a Commercial Sheep Pox Vaccine. Infect. Genet. Evol..

[24] Viarouge C., Breard E., Zientara S., Vitour D., Sailleau C. (2015). Duplex Real-Time RT-PCR Assays for the Detection and Typing of Epizootic Haemorrhagic Disease Virus. PLoS ONE.

[25] Pioz M., Guis H., Calavas D., Durand B., Abrial D., Ducrot C. (2011). Estimating Front-Wave Velocity of Infectious Diseases: A Simple, Efficient Method Applied to Bluetongue. Vet. Res..

[26] Martinelle L., Dal Pozzo F., Thys C., De Leeuw I., Van Campe W., De Clercq K., Thiry E., Saegerman C. (2018). Assessment of Cross-Protection Induced by a Bluetongue Virus (BTV) Serotype 8 Vaccine towards Other BTV Serotypes in Experimental Conditions. Vet. Res..

[27] Fay P.C., Jaafar F.M., Batten C., Attoui H., Saunders K., Lomonossoff G.P., Reid E., Horton D., Maan S., Haig D. (2021). Serological Cross-Reactions between Expressed Vp2 Proteins from Different Bluetongue Virus Serotypes. Viruses.

[28] Santman-Berends I.M.G.A., van Schaik G., Bartels C.J.M., Stegeman J.A., Vellema P. (2011). Mortality Attributable to Bluetongue Virus Serotype 8 Infection in Dutch Dairy Cows. Vet. Microbiol..

[29] Elbers A.R.W., Backx A., Mintiens K., Gerbier G., Staubach C., Hendrickx G., van der Spek A. (2008). Field Observations during the Bluetongue Serotype 8 Epidemic in 2006. II. Morbidity and Mortality Rate, Case Fatality and Clinical Recovery in Sheep and Cattle in the Netherlands. Prev. Vet. Med..

[30] Saegerman C., Mellor P.S., Berkvens D., Pozzo F.D. (2008). Epidémiologie de La Fièvre Catarrhale Ovine En Europe. Fièvre Catarrhale Ovine.

[31] Conraths F.J., Gethmann J.M., Staubach C., Mettenleiter T.C., Beer M., Hoffmann B. (2009). Epidemiology of Bluetongue Virus Serotype 8, Germany. Emerg. Infect. Dis..

[32] Wouda W., Roumen M.P.H.M., Peperkamp N.H.M.T., Vos J.H., van Garderen E., Muskens J. (2008). Hydranencephaly in Calves Following the Bluetongue Serotype 8 Epidemic in the Netherlands. Vet. Rec..

[33] Vinomack C., Rivière J., Bréard E., Viarouge C., Postic L., Zientara S., Vitour D., Belbis G., Spony V., Pagneux C. (2020). Clinical Cases of Bluetongue Serotype 8 in Calves in France in the 2018–2019 Winter. Transbound. Emerg. Dis..

[34] Bulletin Épidémiologique N°35. Réseau Wallon D’Épidémiosurveillance Des Avortements Bovins. ARSIA. https://www.arsia.be/wp-content/uploads/PDF-Arsia-Infos/2025/AI-Janvier-2025-FR.pdf.

[35] Santman-Berends I.M.G.A., Hage J.J., van Rijn P.A., Stegeman J.A., van Schaik G. (2010). Bluetongue Virus Serotype 8 (BTV-8) Infection Reduces Fertility of Dutch Dairy Cattle and Is Vertically Transmitted to Offspring. Theriogenology.

[36] Kirschvink N., Raes M., Saegerman C. (2009). Impact of a Natural Bluetongue Serotype 8 Infection on Semen Quality of Belgian Rams in 2007. Vet. J..

[37] Müller U., Kemmerling K., Straet D., Janowitz U., Sauerwein H. (2010). Effects of Bluetongue Virus Infection on Sperm Quality in Bulls: A Preliminary Report. Vet. J..

[38] Leemans J., Raes M., Vanbinst T., De Clercq K., Saegerman C., Kirschvink N. (2012). Viral RNA Load in Semen from Bluetongue Serotype 8-Infected Rams: Relationship with Sperm Quality. Vet. J..

[39] Martinelle L., Dal Pozzo F., Thiry E., De Clercq K., Saegerman C. (2019). Reliable and Standardized Animal Models to Study the Pathogenesis of Bluetongue and Schmallenberg Viruses in Ruminant Natural Host Species with Special Emphasis on Placental Crossing. Viruses.

[40] Nusinovici S., Seegers H., Joly A., Beaudeau F., Fourichon C. (2012). Quantification and At-Risk Period of Decreased Fertility Associated with Exposure to Bluetongue Virus Serotype 8 in Naïve Dairy Herds. J. Dairy Sci..

[41] Melkleveringen (BCZ CBL). https://bcz-cbl.be/nl/domeinen/melkaanvoer/melkleveringen.

[42] Melkaanvoer en Zuivelproductie Door Zuivelfabrieken (Central Bureau Voor de Statistiek). https://www.cbs.nl/nl-nl/cijfers/detail/7425zuiv.

[43] Cargnel M., Van der Stede Y., Haegeman A., De Leeuw I., De Clercq K., Méroc E., Welby S. (2019). Effectiveness and Cost-Benefit Study to Encourage Herd Owners in a Cost Sharing Vaccination Programme against Bluetongue Serotype-8 in Belgium. Transbound. Emerg. Dis..

[44] Velthuis A.G.J., Saatkamp H.W., Mourits M.C.M., de Koeijer A.A., Elbers A.R.W. (2010). Financial Consequences of the Dutch Bluetongue Serotype 8 Epidemics of 2006 and 2007. Prev. Vet. Med..

[45] Vitour D., Guillotin J., Sailleau C., Viarouge C., Desprat A., Wolff F., Belbis G., Durand B., Bakkali-Kassimi L., Breard E. (2011). Colostral Antibody Induced Interference of Inactivated Bluetongue Serotype-8 Vaccines in Calves. Vet. Res..

[46] van der Sluijs M.T.W., Schroer-Joosten D.P.H., Fid-Fourkour A., Vrijenhoek M.P., Debyser I., Gregg D.A., Dufe D.M., Moulin V., Moormann R.J.M., de Smit A.J. (2012). Effect of Vaccination with an Inactivated Vaccine on Transplacental Transmission of BTV-8 in Mid Term Pregnant Ewes and Heifers. Vaccine.

[47] Leemans J., Hamers C., Chery R., Bibard A., Besancon L., Duboeuf M., Hudelet P., Goutebroze S., Kirschvink N. (2013). Interference of Colostral Antibodies with Response to a Bluetongue Serotype 8 Inactivated Vaccine in Lambs Born from Hyperimmune Ewes. Vaccine.

[48] Farooq U., Ahmed S., Liu G., Jiang X., Yang H., Ding J., Ali M. (2024). Biochemical Properties of Sheep Colostrum and Its Potential Benefits for Lamb Survival: A Review. Anim. Biotechnol..

[49] EFSA AHAW Panel (EFSA Panel on Animal Health and Welfare) (2017). Scientific opinion on bluetongue: Control, Surveillance and Safe Movement of Animals. EFSA J..

[50] Kirkland P.D., Melville L.F., Hunt N.T., Williams C.F., Davis R.J. (2004). Excretion of Bluetongue Virus in Cattle Semen: A Feature of Laboratory-Adapted Virus. Vet. Ital..

[51] EFSA AHAW Panel (EFSA Panel on Animal Health and Welfare) (2011). Scientific Opinion on Bluetongue Serotype 8. EFSA J..

[52] De Clercq K., Vandaele L., Vanbinst T., Riou M., Deblauwe I., Wesselingh W., Pinard A., Van Eetvelde M., Boulesteix O., Leemans B. (2021). Transmission of Bluetongue Virus Serotype 8 by Artificial Insemination with Frozen–Thawed Semen from Naturally Infected Bulls. Viruses.

[53] Wrathall A.E., Simmons H.A., Van Soom A. (2006). Evaluation of Risks of Viral Transmission to Recipients of Bovine Embryos Arising from Fertilisation with Virus-Infected Semen. Theriogenology.

[54] Haegeman A., Vandaele L., De Leeuw I., Oliveira A.P., Nauwynck H., Van Soom A., De Clercq K. (2019). Failure to Remove Bluetongue Serotype 8 Virus (BTV-8) From in Vitro Produced and in Vivo Derived Bovine Embryos and Subsequent Transmission of BTV-8 to Recipient Cows After Embryo Transfer. Front. Vet. Sci..

[55] Linden A., Grégoire F., Nahayo A., Hanrez D., Mousset B., Massart L., de Leeuw I., Vandemeulebroucke E., Vandenbussche F., de Clercq K. (2010). Bluetongue Virus in Wild Deer, Belgium, 2005–2008. Emerg. Infect. Dis..

[56] Mauroy A., Guyot H., De Clercq K., Cassart D., Thiry E., Saegerman C. (2008). Bluetongue in Captive Yaks. Emerg. Infect. Dis..

[57] Caballero-Gómez J., Cano Terriza D., Pujols J., Martínez-Nevado E., Carbonell M.D., Guerra R., Recuero J., Soriano P., Barbero J., García-Bocanegra I. (2022). Monitoring of Bluetongue Virus in Zoo Animals in Spain, 2007–2019. Transbound. Emerg. Dis..

[58] Martinelle L., Haegeman A., Lignereux L., Chaber A.-L., Dal Pozzo F., De Leeuw I., De Clercq K., Saegerman C. (2022). Orbivirus Screening from Imported Captive Oryx in the United Arab Emirates Stresses the Importance of Pre-Import and Transit Measures. Pathogens.

