# Supplementary material for: Impact of BTV-3 Circulation in Belgium in 2024 and Current Knowledge Gaps Hindering an Evidence-Based Control Program
Source: Viruses. 2025 Apr 3;17(4):521. doi: 10.3390/v17040521 (PMC12031096; doi:10.3390/v17040521)
Supplement: Supplementary file 1 [file viruses-17-00521-s001.zip › Supplementary material_Article BTV.pdf]

Supplementary materials

Table S1 : Excess number of dead animals recorded in 2024 compared to the mean of the years 2021 to 2023 (%) in the different cattle categories.

	Calf up to 10 kg	Calf 25 kg	Calf 50 kg	Bovine 150 kg	Bovine 300 kg	Bovine 550 kg	Bovine 800 kg	Bovine Total
January	8,6	33,8	-6,3	13,3	4,1	8,2	26,6	**2,0**
February	9,4	19,6	-14,4	9,9	-2,8	3,4	20,7	**-5,7**
March	-2,3	8,6	-25,0	-13,1	-11,2	-12,6	18,6	**-17,5**
April	9,7	21,2	-10,8	-8,9	-10,1	-0,6	40,3	**-5,4**
May	12,0	29,8	-3,8	-5,1	7,6	4,2	38,3	**2,0**
June	2,7	4,9	-20,1	-14,6	-7,2	-9,0	11,0	**-13,7**
July	26,0	54,6	0,9	21,7	33,6	11,4	44,5	**12,4**
Augustus	37,7	87,5	23,9	18,0	53,7	57,8	80,6	**38,3**
September	127,6	121,7	28,7	19,9	66,9	38,4	63,5	**45,1**
October	152,9	84,6	13,7	29,2	50,2	13,3	25,7	**30,0**
November	92	65	-6	9	17	-6	1	**6**
December	68	61	0	9	23	-11	-2	**8**
Global year	45,6	49,4	-2,9	7,8	17,6	7,6	30,8	**7,4**
